# A review of insect parasitoids associated with *Lobesia botrana* (Denis & Schiffermüller, 1775) in Italy. 1. Diptera Tachinidae and Hymenoptera Braconidae (Lepidoptera, Tortricidae)

**DOI:** 10.3897/zookeys.647.11098

**Published:** 2017-01-23

**Authors:** Pier Luigi Scaramozzino, Augusto Loni, Andrea Lucchi

**Affiliations:** 1 Dept of Agriculture, Food & Environment, University of Pisa – Via del Borghetto, 80 - 56124 Pisa, Italy

**Keywords:** Biological control, braconid wasps, European grapevine moth, natural enemies, tachinid flies

## Abstract

This paper is aimed to summarize the information available on the parasitoid complex of the European Grapevine Moth (EGVM), *Lobesia botrana* (Denis & Schiffermüller, 1775) (Lepidoptera Tortricidae) in Italy. The list is the result of the consultation of a vast bibliography published in Italy for almost two hundred years, from 1828 to date. This allowed the clarification and correction of misunderstandings and mistakes on the taxonomic position of each species listed.

In Italy the complex of parasitoids detected on EGVM includes approximately 90 species belonging to ten families of Hymenoptera (Braconidae, Ichneumonidae, Chalcididae, Eulophidae, Eupelmidae, Eurytomidae, Pteromalidae, Torymidae, Trichogrammatidae, and Bethylidae) and one family of Diptera (Tachinidae). This paper deals with EGVM parasitoids of the families Tachinidae (Diptera) and Braconidae (Hymenoptera). Only two species of Tachinidae are associated to EGVM larvae in Italy, *Actia pilipennis* (Fallen) and *Phytomyptera nigrina* (Meigen), whereas the record of *Eurysthaea scutellaris* (Robineau-Desvoidy) is doubtful. Moreover, 21 species of Braconidae are reported to live on EGVM, but, unfortunately, eight of them were identified only at generic level. *Bracon mellitor* Say has been incorrectly listed among the parasitoids of *Lobesia botrana*. Records concerning *Ascogaster rufidens* Wesmael, *Meteorus* sp., *Microgaster rufipes* Nees, and *Microplitis tuberculifer* (Wesmael) are uncertain.

## Introduction

The European Grapevine Moth (EGVM), *Lobesia botrana* (Denis & Schiffermüller, 1775) (Lepidoptera, Tortricidae) is an important pest in the grape-growing regions of Europe, the Middle East, northern and western Africa and southern Russia (CABI 2016a), whereas its occurrence in Japan has been invalidated (Bae and Komai 1991). This species was accidentally introduced in North and South America. It was found for the first time in California in 2009 (Varela et al. 2010, Gilligan et al. 2011, Ioriatti et al. 2012), in Chile in 2008 (Gonzalez 2010, Ioriatti et al. 2012) and in Argentina in 2010 (SENASA 2010, Ioriatti et al. 2012, SENASA 2016).

EGVM massively appeared in the wine-growing areas of southern Europe (France, Italy, the Iberian Peninsula) at the end of 1800. A century before, the species had been named but not described by Denis and Schiffermüller (1775 and 1776) as *Tortrix botrana*.

Later on, the moth was described by Jacquin (1788), OG Costa (1840), Milliere (1865) and Dufrane (1960) under different names.

The taxonomic history of EGVM is rather complicated; over time the species has been attributed to various genera or it has been misinterpreted as different species, generating confusion in biological data and at the synonymic level. In the “Datasheet Report” for European Grapevine Moth of CABI (2016a) this confusion is still present and the list of “Other Scientific Names” shows synonymies, mainly due to misinterpretation, which are no longer valid: *Tortrix reliquana* Hübner, 1825 (= *Lobesia reliquana*) is a valid species; *Penthina vitivorana* Packard, 1869 is synonym of *Paralobesia viteana* (Clemens, 1860); *Tinea* “*premixtana*” is a wrong spelling for *Tortrix* “*permixtana*” Hübner, 1796, probably the *Tortrixpermixtana auct. nec* Denis & Schiffermüller, 1775, which is synonymous with the aforementioned *Lobesia reliquana* (Hübner) (cf. Brown 2005, Fauna Europaea). Finding papers with the original reports, in the continuous transfer from a publication to another, has required a lot of work and the appreciated help of various colleagues. The continuous progress of the taxonomic knowledge and the numerous changes that have been and are still proposed, required supervision and updating of the names of the species attributed by the former authors, especially those who published their data before the second half of 1900.

Since its first record, EGVM had been associated with the grapevine (Denis and Schiffermüller 1775). Subsequently, its biology and its damage to the grapevine was defined (Jacquin 1788, Kollar 1837 [1840]). It is only in the second half of the 19^th^ century that the species fully showed its aggressiveness, alarming wine-makers and attracting the interest of applied entomologists. In Italy the first report of *Lobesia botrana* is attributed to Oronzo Gabriele Costa (1828), who found the moth in the Otranto surroundings (Apulia) on *Olea europaea* L. inflorescences, and classified the species as *Noctua romana*, later replaced by *Noctua romaniana* (OG Costa 1840, A. Costa 1857, 1877, Del Guercio 1899, Silvestri 1912, Tremewan 1977). In 1849 Semmola described the damage on the grapevine in the Vesuvian region of Naples (A. Costa 1857). In 1869 Levi, in a paper on the grape moth “Tortrix Uvae” or “uvana”, *Eupoecilia ambiguella*, which heavily infested vineyards near Gorizia, mentioned the presence of three larvae of a second “grape worm”, which he found before the harvest, and whose larva was characterized by a “… *more lively and spirited temperament that made him squirming and slipping from the hands like an eel*”, and which he attributed to *Tortrix vitisana* that is today a synonym for *Lobesia botrana*. He recalls the subject a few years later (Levi 1873), with news on parasitoids of *Eupoecilia ambiguella*. At the same time, Dei (1873) assigned to *Lobesia botrana* the liability of the heavy damage caused to the grapevine in Trieste district and in other parts of Italy (Ioriatti and Anfora 2007). Also Ghiliani (1871) and De Stefani (1889) mentioned the species (as *Lobesia permixtana*) for its damage to grape.

With regard to the grape moth parasitoids, Camillo Rondani (1871-1878) reported only one Ichneumonid, *Pimpla instigator* Fabricius, 1793, living on *Cochylis roserana* Frölich, 1828 (= *Eupoecilia ambiguella*), but did not mention *Lobesia botrana*.

In 1899, Del Guercio described in detail the morphology and the behavior of EGVM, providing the first list of seven parasitoids obtained from larvae and pupae collected in the vineyards of Tuscany. In a paper dealing with Italian Chalcidoidea, Masi (1907) reported three species emerged from EGVM, one of which he described as *Dibrachys affinis*. Later on, he named another Chalcidoid as *Elachistus affinis*, also obtained from EGVM (Masi 1911).

At the time when Paul Marchal in France was publishing an important work on EGVM (1912), in Italy Giulio Catoni (1910-1914) and Filippo Silvestri (1912) carried out their investigations, in Trentino-South Tyrol and Campania (Portici-Naples) respectively, publishing interesting information on EGVM.

With the impressive collections of pupae of the first spring-summer generation and of the overwintering second generation, Catoni collected EGVM and EGBM individuals in varying proportions, although frequently EGVM was more abundant. The purpose of his investigations was to provide a valid argument to declare as mandatory the “autumnal application of bands and rags to the vine stems” with the aim to collect the migrating larvae, prevent moth emerging and allow parasitoid spreading. From these pupae Catoni obtained 15 species of parasitoids (Catoni 1914). Silvestri (1912) described rather accurately the morphology and habits of EGVM, providing comprehensive information of 26 species of parasitoids. These important contributions are followed by the list of EGVM parasitoids reported in Italy until the year 1911 by Gustavo Leonardi (1925), who mentioned 21 species, and by Francesco Boselli (1928), who listed 42 species from 1911 to 1925. The results of Catoni and Silvestri describing EGVM parasitoids were then mentioned by Stellwag (1928) and reviewed by Thompson (1946).

After a long period of time of nearly 70 years, in which the essays on EGVM parasitoids were very rare, in the 1990s Marchesini and Dalla Montà (1994) published a long list of parasitoids associated to EGVM in the Veneto vineyards. With the introduction of the IPM (Integrated Pest Management) principles, the role of natural enemies was more and more emphasized and the interest for the EGVM parasitoids in Italy came back, and the investigations - though occasional - were never interrupted to date.

The other vine moth, *Eupoecilia ambiguella* (Hübner, 1796) (European Grape Berry Moth, EGBM) was recognized as the major grape berry pest in Europe until the 1920s (Berlese 1900, Solinas 1962, Bovey 1966). More recently and in many areas, it has been gradually replaced by *Lobesia botrana*. The shift started in the Mediterranean Basin and is now extending - for climatic reasons - to Central Europe, where populations of EGVM and EGBM overlap.

EGVM larvae feed on grapevine flowers and berries and on a number of other plants growing in warm-dry environments. Its host range includes approximately 40 species belonging to 27 families (Coscollá 1997). The spurge flax *Daphne gnidium* L. (Malvales Thymelaeaceae) is considered as its original host plant (Marchal 1912, Balachowky and Mesnil 1935, Bovey 1966, Lucchi and Santini 2011, Tasin et al. 2011, CABI 2016a, Lucchi et al. in press). However, EGVM is frequently associated with other hosts in habitats where suitable host plants occur. These include olive tree inflorescence (O. Costa 1828 and 1840, Tzanakakis and Savopoulou-Soultani 1973, Stoeva 1982, Savopoulou-Soultani et al. 1990, Coscollá 1997, Stavridis and Savopoulou-Soultani 1998, Thiéry 2005, Roditakis in Ioriatti et al. 2011), Virginia creeper (Stoeva 1982, Coscollá 1997, Thiéry 2005, CABI 2016a), jujube (Bovey 1966, Coscollá 1997, Thiéry 2005, CABI 2016a), rosemary (Bovey 1966, Coscollá 1997, Thiéry 2005, Roditakis in Ioriatti et al. 2011, CABI 2016a), arbutus (Coscollá 1997, Thiéry 2005, CABI 2016b) evergreen clematis (Stoeva 1982, Coscollá 1997, Thiéry 2005, CABI 2016a), dogwood (Stoeva 1982, Coscollá 1997, Thiéry 2005, CABI 2016a), ivy (Bovey 1966, Coscollá 1997), currant (Bovey 1966, Stoeva 1982, Coscollá 1997, Thiéry 2005, CABI 2016a).

Larval feeding on green and ripe berries of grapevine allows the infection by various microorganisms that frequently results in bunch rots (Fermaud and Le Menn 1989), making this leafroller the most economically important pest of grapevine in the wine-growing areas, worldwide (Ioriatti et al. 2011). The natural enemy assemblage of *Lobesia botrana* varies considerably both in time and space. It includes entomopathogenic fungi, bacteria and baculovirus, together with a long list of arthropod predators as spiders and insects belonging to Dermaptera, Hemiptera, Neuroptera, Diptera and Coleoptera, as well as parasitoids (Marchesini and Dalla Montà 1994, Coscollá 1997, Ioriatti et al. 2011, Sentenac 2012, CABI 2016a). The complex of insect parasitoids feeding on *Lobesia botrana* in Europe includes mainly Hymenoptera Ichneumonoidea, Chalcidoidea, Bethyloidea and few species belonging to Diptera (Tachinidae) (Martinez et al. 2006).

Extensive scientific efforts are still needed to develop biological control as an effective solution for practical use in the field. Egg parasitoids of the genus *Trichogramma* have been mass-released in a inundative strategy with variable results (Castaneda-Samayoa et al. 1993, Hommay et al. 2002, Ibrahim 2004), though they can be frequently found in cultivated and natural environments (Barnay et al. 2001, Ibrahim 2004, Lucchi et al. 2016). The pteromalids *Dibrachys affinis* Masi and *Dibrachys cavus* (Walker) are gregarious generalist larval-pupal parasitoids of Lepidoptera, Diptera and Hymenoptera that can be readily reared in the laboratory. However, due to the lack of host specificity and because they can also behave as hyperparasites, they have not considered as good candidates for biological pest control. An ichneumonid species, *Campoplex capitator* Aubert, is known as the most frequent and efficient parasitoid of EGVM in European vineyards. It is a larval parasitoid that has been regarded as the best candidate for future EGVM biological control programs. Substantial releases have not taken place because of the difficulties associated with artificially mass-rearing of the species (Thiéry and Xuéreb 2004).

The limited knowledge of the field efficacy of EGVM natural enemies has recently come to light for the questions raised in this regard by some American entomologists, who persistently questioned the potential of the biological control for EGVM management (Varela et al. 2010). In this respect, there is an urgent need to check the existing literature with the aim to critically revise the taxonomic nomenclature, assigning to each species its valid name and assessing their potential as biocontrol agents.

We fully agree with what was written by William Robin Thompson in the “Catalogue of Parasites and Predators of Insects Pests” (published under his direction in several volumes from 1943 to 1972) concerning the introduction of natural enemies of insect pests accidentally introduced in a new country: “… it is necessary to know the identity and habits for the parasites and predators attacking the pest in its native home. The name and habits of the natural enemies of many pests are recorded in the literature, but it is usually a very difficult and tedious task to assemble the information. A comprehensive list or catalogue of the predators of injurious insects, with the reference to the original papers in which they were recorded is, therefore, one of the fundamental necessities in biological control work.” (Thompson 1943).

Among the difficulties that can arise when compiling these lists, Thompson suggests mainly the “inaccuracy in observation, rearing work and identification contained in the works of former authors, which greatly limits their practical value.” Many past mistakes of unusual parasitoid species associated to EGVM in Italy might be due to a poor lab management of the field collection. We should take into consideration that Catoni (1910-14), Ruschka and Fulmek (1915) and, more recently, Colombera et al. (2001), have proposed lists of parasitoids obtained by clusters where the two vine moths were possibly present, without indicating from which individual each parasitoid was obtained. Nevertheless, it is also true that often the two tortricids may share the same parasitoids (see e.g.: Villemant et al. 2012, tableau 2.9). Those parasitoids have often a fairly broad host range and can attack suitable hosts living in the same environment, on the same plant, and even on the same cluster (Loni et al. 2012, Scaramozzino et al. in press).

Over time, the observations and the rearing techniques have been refined and rarely constitute a serious obstacle to this type of investigation. On the other hand, there is still a great difficulty in parasitoid and predator identification, which is intrinsic to the vastness and complexity of the taxonomic groups to which they belong.

This paper deals with Diptera Tachinidae and Hymenoptera Braconidae and aims to be the first contribution of a revised and updated list of the Italian parasitoids of *Lobesia botrana*.

## Materials and methods

### *Lobesia botrana* and its parasitoids in Italy

The Italian records on EGVM parasitoids are a fragmented patchwork. This paper includes data from fewer than half of the Italian regions (nine of 20), and most of these data come from the northern part of Italy (Trentino-South Tyrol 37 species, Veneto 31 species, Piedmont 25 species) followed by Sardinia (22 species) Tuscany (20 species), Campania (19 species), Apulia (7 species), and Sicily and Umbria (1 species). An important part of the information (e.g.: Trentino-South Tyrol, Campania, Tuscany, Umbria and Sicily) comes from works published between the end of 19^th^ and early 20^th^ centuries, and some specific identifications are not responding to current taxonomic criteria, so requiring an accurate revision.

Most of the data result from studies conducted in the vineyards (approx. 85 species recorded in 29 papers) and some from the spurge flax (*Daphne gnidium* L.) in natural or semi-natural environments (appox. 15 species and 6 papers). In some contributions, mostly focused on general aspects, such as for example the grapevine protection from EGVM attacks, the reports on parasitoids are marginal and not very consistent.

The origin, quality and consistency of the data are not uniform and reflect the absence, in certain regions, of people with the necessary scientific knowledge and skill to carry out this type of investigation.

The list of parasitoid species feeding on *Lobesia botrana* in Italy was drawn up using all the papers published on the subject, both in Italy (Table 1) and worldwide. We also revised the lists of parasitoids compiled by Thompson (1943), Coscollá (1997), Hoffman and Michl (2003) and by CABI (2016b). The names of the species have been verified and updated by following the on-line “Home of Ichneumonoidea” and flash-drive Taxapad databases of Yu et al. (1997-2012, 2012), Noyes (2014) and the Fauna Europaea (de Jong et al. 2014).

**Table 1.** References consulted for the compilation of the parasitoid list of the European grapevine moth in Italy. See references for the full bibliographic citation. The numbers on the left are the same as in Table 2.

Number	Authors
1	Bagnoli B, Lucchi A (2006)
2	Barbieri R, Cavallini G, Pari P, Guardigni P (1992)
3	Baur H (2005)
4	Boselli F (1928)
5	Caotni G (1910)
6	Catoni G (1914)
7	Cerretti P, Tschornig H-P (2010)
8	Colombera S, Alma A, Arzone A (2001)
9	Dalla Montà L, Marchesini E (1995)
10	Del Guercio G (1899)
11	Delrio G, Luciano P, Prota R (1987)
12	Forti D (1991)
13	Laccone G (1978)
14	Leonardi G (1925)
15	Loni A, Samartsev KG, Scaramozzino PL, Belokobylkij SA, Lucchi A (2016)
16	Lozzia GC, Rigamonti EI (1991)
17	Lucchi A, Santini L (2011)
18	Lucchi A, Scaramozzino PL, Michl G, Loni A, Hoffmann C (2016)
19	Luciano P, Delrio G, Prota R (1988)
20	Marchesini E, Dalla Montà L (1994)
21	Marchesini E, Dalla Montà L (1998)
22	Marchesini E, Dalla Montà L, Sancassani GP (2006)
23	Masi L (1907)
24	Masi L (1911)
25	Moleas T (1979)
26	Moleas T (1995)
27	Nobili P, Correnti A, Vita G, Voegelé J (1988)
28	Nuzzaci G, Triggiani O (1982)
29	Pinna M, Gremo F, Scaramozzino PL (1989)
30	Roat C, Forti D (1994)
31	Ruschka F, Fulmek L (1915)
32	Scaramozzino PL, Loni A, Lucchi A, Gandini L (In press)
33	Silvestri F (1912)
34	Stellwaag F (1928)
35	Zangheri S, Dalla Montà L, Duso C (1987)

Various names are not related to any species currently known and are considered “nomina dubia”, while some misspellings have been amended. The list contains the names used by the different authors in their publications and those updated according to the sources mentioned above. Names no longer valid are preceded by a dot and are followed by the name of the authors who used them. Within the list, the species are divided by Order and Family and sorted alphabetically. Valid names are in bold. Synonyms, misspellings, combinations other than those valid today, are in a smaller font and show in square brackets the valid name. The papers examined and included in the list are sorted alphabetically and consecutively numbered. These numbers are shown in the table, in the columns of the main geographical areas in which Italy can be divided: northern Italy (indicated by NORTH and including the Regions of Valle d’Aosta, Piedmont, Liguria, Lombardy, Trentino-South Tyrol, Veneto, Friuli-Venezia Giulia and Emilia-Romagna); Central Italy (shown with CENTER and including Tuscany, Marche, Umbria, Lazio and Abruzzo), southern Italy (indicated with SOUTH, including Campania, Molise, Apulia, Basilicata and Calabria), Sicily and Sardinia. In two separate columns we indicated if the record is earlier or later than 1970. If the species has been recorded before and after that date, it is shown on both columns.

## Results

The complex of parasitoids detected on EGVM in Italy includes some 90 species belonging to ten families of Hymenoptera (Braconidae, Ichneumonidae, Chalcididae, Eulophidae, Eupelmidae, Eurytomidae, Pteromalidae, Torymidae, Trichogrammatidae and Bethylidae) and one family of Diptera (Tachinidae). More than fifty species belong to Ichneumonidae, followed by Braconidae with 21 species, Eulophidae eight species, Trichogrammatidae six species, and Pteromalidae five species. All the other families are represented by one or two species. The parasitoids of EGVM, belonging to the families Tachinidae (Diptera) and Braconidae (Hymenoptera), reported in Italy by various authors (see Table 1) are listed in Table 2.

**Table 2.** List of Tachinidae (Diptera) and Braconidae (Hymenoptera) parasitoids of *Lobesia botrana* reported in Italy.

Order-family / subfamily / species	subfamily	<1970	>1970	NORTH	CENTER	SOUTH	SICILY	SARDINIA
**DIPTERA** **TACHINIDAE**																
***Actia pilipennis*** (Fallen, 1810)	Tachininae		•		32			
• *Discochaeta hyponomeutae* Rond. [= ***Eurysthaea scutellaris***]								
***Eurysthaea scutellaris*** (Robineau-Desvoidy, 1848)			?	Forti in Coscollá 1997 (as *Dischocaeta hyponomeutae*)				
***Phytomyptera* sp.**	Tachininae		•			27		
***Phytomyptera nigrina*** (Meigen, 1824)	Tachininae	•[4, 14: as *Phytomyptera nitidiventris*] 14 (as Phytomyptera nitidiventris var. unicolor Rond.)	•	3, 8, 20, 21, 23	1, 10, 33 (as *Phytomyptera nitidiventris* and Phytomyptera nitidiventris var. unicolor), 36	13, 28, 33 (as *Phytomyptera nitidiventris* and Phytomyptera nitidiventris var. unicolor)	25 (as *Phytomyptera nitidiventris* and Phytomyptera nitidiventris var. unicolor)	19
• *Phytomyptera nitidiventris* Rond. [= ***Phytomyptera nigrina***]								
• Phytomiptera nitidiventris var. unicolor Rond. [= ***Phytomyptera nigrina***]								
• *Phytomiptera unicolor* Rond. [= ***Phytomyptera nigrina***]								
**HYMENOPTERA**																
**BRACONIDAE**								
***Agathis* sp.** Latreille, 1804	Agathidinae		•					11
***Agathis malvacearum*** Latreille, 1805	Agathidinae		•			25, 26		
***Apanteles* sp.** Forster, 1862	Microgastrinae		•			28		19
***Apanteles albipennis*** (Nees, 1834)	Microgastrinae		•			13		
***Aleiodes* sp.** Wesmael, 1838	Rogadinae		•			28		
***Ascogaster quadridentata*** Wesmael,1835	Cheloninae		•	20, 21, 22	1			19
***Ascogaster rufidens*** Wesmael, 1835	Cheloninae	• 4				33		
***Bassus linguarius*** (Nees, 1812)	Agathidinae		•			28		
**Bracon (Glabrobracon) admotus** Papp, 2000	Braconinae		•		15, 32 (as *Bracon* spp.)			
***Bracon mellitor*** Say, 1836	Braconinae	• 14 (as *Bracon vernoniae*)						
• *Bracon vernoniae* Ashm. [= ***Bracon mellitor***]								
***Chelonus* sp.** Panzer, 1806	Cheloninae		•					11
***Colastes* sp.** Haliday, 1833	Exothecinae		•	8				
***Habrobracon* sp.** Ashmead, 1895	Braconinae		•			26		11
• *Habrobracon brevicornis* Wesmael [= ***Habrobracon hebetor***]								
***Habrobracon concolorans*** (Marshall, 1900)	Braconinae		•		15, 32 (as *Bracon* spp.)			
***Habrobracon hebetor*** (Say, 1836)	Braconinae	• 4 (as *Habrobracon* sp.)	•		15, 32 (as *Bracon* spp.)	25, 33 (as *Habrobracon* sp.), Goidanich, 1934	33 (as *Habrobracon* sp. from *Ephestia elutella*)	
***Habrobracon pillerianae*** Fischer, 1980	Braconinae		•		15, 32 (as *Bracon* spp.)			
***Meteorus* sp.** Haliday,1835	Euphorinae	• 4				33		
• *Microbracon brevicornis* Wesmael [= ***Habrobracon hebetor***]		• Thompson, 1946						
• *Microgaster globata* (Linnaeus, 1758) [= ***Microgaster rufipes***]								
***Microgaster rufipes*** Nees, 1834	Microgastrinae	•		6 (as *Microgaster globata*)				
***Microplitis* sp.** Foerster, 1862	Microgastrinae		•	8, 20, 21, 22				
***Microplitis tuberculifer*** (Wesmael, 1837)	Microgastrinae	• 4 (as *Microplitis tuberculifera*)		6, 31				
***Therophilus tumidulus*** (Nees, 1812)	Agathidinae		•					19 (as *Microdus tumidulus*)

Table explanation. First column shows: 1- Order and Family to which the parasitoid belongs (e.g.: DIPTERA Tachinidae), 2 - Valid specific names of parasitoids in bold italics followed by the author who described the species and the year of description. The author’s name and the year are in parenthesis if the species is assigned to a genus different from the original description (e.g.: *Itoplectis alternans* (Gravenhorst, 1829) was described and included in 1829 by Gravenhorst in the genus *Pimpla* while it is now assigned to the genus *Itoplectis*), 3 - Names that are in synonymy, or which relate to combinations genus-species no longer valid and to incorrect spellings, as found in the works cited in the references. These names are preceded by a black dot and are followed by the valid name in bold and in square brackets to which it refers in the list (e.g.: *Phytomyptera nitidiventris* Rond. [= *Phytomyptera nigrina*]). Second column includes, only for the valid species, the relating subfamily. Third column titled “<1970”, are indicated with a dot the valid species recorded before that date. Fourth column titled “> 1970”, are indicated with a dot the valid species recorded after that date. Columns “North”, “Center”, “South”, “Sicily” and “Sardinia” the records that refer to a specific area are shown by a number (which refers to the work mentioned in the “references”), with in parenthesis the name used in the message if it differs from that of the valid species [e.g.: 4 (as *Phytomyptera nitidiventris*)]. If there are several papers that use the same name for a species that is no longer valid, the reference numbers and the invalid names are included in square brackets (e.g.: [4, 14: as *Phytomyptera nitidiventris*]).

### Order: DIPTERA

#### Family: TACHINIDAE

##### Subfamily: Tachininae

###### Actia pilipennis

Taxon classificationAnimaliaDipteraTachinidae

(Fallen, 1810)

Fig. 1

Actia pilipennis Scaramozzino et al. (in press).

####### Italian distribution of reared parasitoids.

Tuscany: Scaramozzino et al. (in press).

####### Distribution.

Palearctic species widely distributed, present, with few exceptions, all over Europe; to the east it reaches the Kuril Islands and Japan through southern Siberia and Mongolia (Andersen 1996).

####### Host range.

It is a rather polyphagous species: little more than fifteen hosts are known, mostly belonging to the family Tortricidae (Mesnil 1963, CABI 2016b). Martinez (2012) points out that this species has been obtained by *Sparganothis pilleriana* (Denis & Schiffermüller, 1775), another important tortricid pest of the grapevine, but, curiously, it has not been found on the European grapevine moth yet. Recently, Delbac et al. (2015) obtained a single specimen of this tachinid fly from *Lobesia botrana* in a Bordeaux vineyard. Unlike *Phytomyptera nigrina* (see below), in this case the maggot of *Actia pilipennis* abandons the dead caterpillar and pupate nearby.

####### Ecological role.

During a research carried out in the natural reserve of Migliarino-San Rossore-Massaciuccoli, Pisa), we have obtained quite often specimens of this Tachinid from larvae of the three generations of EGVM and from larvae of *Cacoecimorpha pronubana* (Hübner, 1799), both living on the shoot tips of *Daphne gnidium* (Scaramozzino et al. in press). In the natural reserve, the species has been raised in small number by EGVM from 2012 to 2015. In 2014 the overall rate of parasitism was quite low, not even reaching 1%.

**Figure 1.**
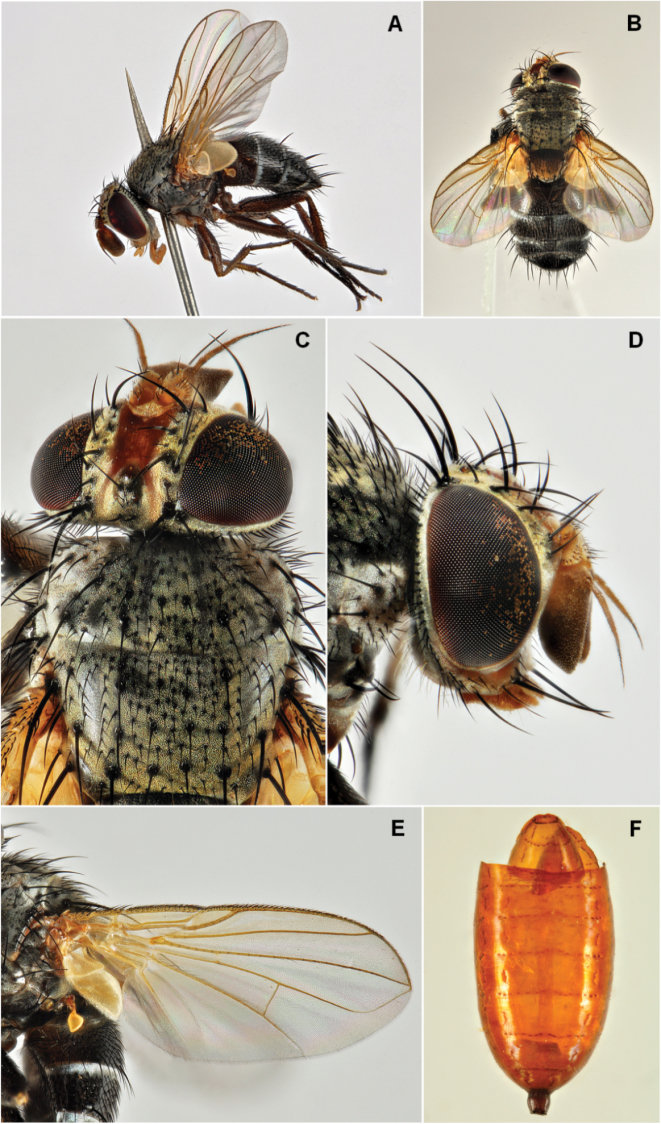
*Actia pilipennis* (Fallen, 1810), female. **A** habitus, lateral view **B** habitus, dorsal view **C** head and anterior part of thorax, dorsal view **D** head, lateral view **E** wing **F** opened puparium.

###### Phytomyptera sp.

Taxon classificationAnimaliaDipteraTachinidae

Phytomyptera sp. Moleas 1995; Coscollá 1997

####### Note.

Very probably the species reported by Moleas (1995) belongs to *Phytomyptera nigrina* (see below): Cerretti (2010) cited six *Phytomyptera* species from Italy, but only *Phytomyptera nigrina* was associated to *Lobesia botrana*.

###### Phytomyptera nigrina

Taxon classificationAnimaliaDipteraTachinidae

(Meigen, 1824) (Pn)

Fig. 2

Phytomyptera nigrina Laccone 1978, Nuzzaci and Triggiani 1982, Luciano et al. 1988, Marchesini and Dalla Montà 1992, 1994, 1998, Coscollá 1997, Colombera et al. 2001, Baur 2005, Marchesini et al. 2006, Bagnoli and Lucchi 2006, Martinez et al. 2006, Cerretti and Tschorsnig 2010, Scaramozzino et al. (in press).
Phytomyptera unicolor Rond.: Del Guercio 1899
Phytomyptera nitidiventris Rond.: Silvestri 1912, Catoni 1914, Leonardi 1925, Boselli 1928, Stellwaag 1928, Thompson 1946
Phytomyptera nitidiventris var. unicolor Rond.: Leonardi 1925
Phytomyptera spp. Moleas 1995, Coscollá 1997

####### Italian distribution of reared parasitoids.

Apulia: Nuzzaci and Triggiani 1982, Laccone 1978

Campania: Silvestri 1912 (Portici, Nola)

Piedmont: Colombera et al. 2001, Baur 2005

Sardinia: Luciano et al. 1988

Tuscany: Del Guercio 1899, Bagnoli and Lucchi 2006, Scaramozzino et al. (in press)

Umbria: Silvestri 1912 (Bevagna)

Veneto: Marchesini and Dalla Montà 1992, 1994, 1998, Marchesini et al. 2006

Emilia-Romagna: Baur 2005 (Bologna, leg. Campadelli)

####### Distribution.

North Central and South Europe, Russia North West, Ukraine (Fauna Europaea)

####### Host range.

Larval endophagous koinobiont parasitoid, *Phytomyptera nigrina* (see Tab. 3) recurs very often in all researches conducted in Italy on parasitoids of *Lobesia botrana*.

**Table 3.** *Phytomyptera nigrina*: percentages of parasitism on the European Grapevine Moth reported in Italy by different authors.

Author/s, publication year	Italian Region	Host plant	Year	1^st^ generation (antophagous)	2^nd^ generation (carpophagous)	3^rd^ generation (carpophagous)
Colombera et al. 2001	Piedmont	grapevine	1998	17.3	0	does not occur
Colombera et al. 2001	Piedmont	grapevine	1999	6.5	(2 specimens)	does not occur
Laccone 1978	Apulia / Cerignola	grapevine	1978	26.08	11,4 / 12,4 / 14,7	0
Marchesini et al. 1994	Veneto/ Pernumia (PD)	grapevine	1989	0	1,76	0
Marchesini et al. 1994	Veneto/ Pernumia (PD)	grapevine	1990	0	0,23	0
Marchesini et al. 1994	Veneto/ Pernumia (PD)	grapevine	1991	0	0,97	0
Marchesini et al. 1994	Veneto/ Colognola (VR)	grapevine	1990	0.36	6,72	0
Marchesini et al. 1994	Veneto/ Colognola (VR)	grapevine	1991	1	0	0
Marchesini et al. 1994	Veneto/ Colognola (VR)	grapevine	1992	0	0,48	0
Marchesini et al. 1994; Marchesini et al. 2006	Veneto/ Valpolicella (VR)	grapevine	1992 (1)	0 / 0.64	0,48 / 2,14	0 / 0
Marchesini et al. 2006	Veneto	grapevine	2000 (2)		14,6 / 4,4	0 / 0
Marchesini et al. 2006	Veneto	grapevine	2001 (2)	0 / 0	1,0 / 0,8	0 / 0
Nuzzacci and Triggiani 1982	Apulia	Daphne gnidium	1979–1982	?	?	30
Luciano et al. 1988	Sardinia	Daphne gnidium	1986–87	25-24,1	?	7,1-0

(1) Data obtained in vineyards treated with both BT (*Bacillus thuringiensis*) and MP (methyl-parathion)

(2) Data obtained in vineyards with chemical defense or biological defense.

This insect is associated to 29 species of Lepidoptera: Pterophoridae, Pyralidae, Sesiidae, Yponomeutidae and various genera and species of Tortricidae, included *Eupoecilia ambiguella*.

**Figure 2.**
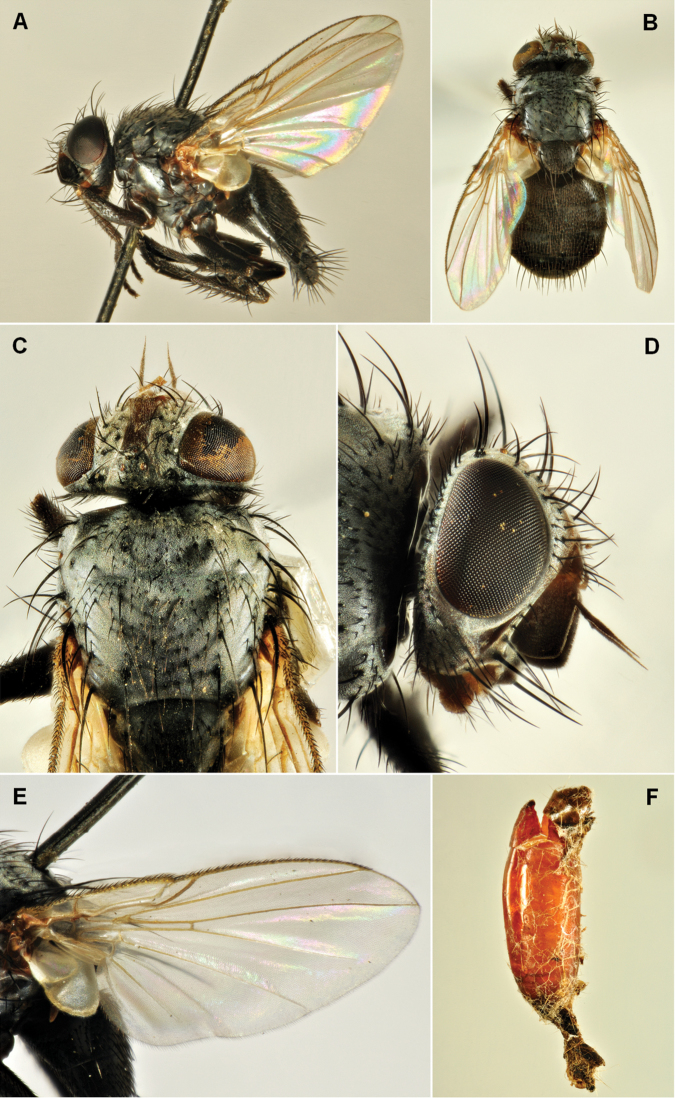
*Phytomyptera nigrina* (Meigen, 1824), female. **A** habitus, lateral view **B** habitus, dorsal view **C** head and anterior part of thorax, dorsal view **D** head, lateral view **E** wing **F** opened puparium tight to the skin of the EGVM dead larva.

Among the Tachinidae living on the vine moths, Pn shows the lowest number of hosts. For more details, see Martinez et al. (2006) and with regard to the hosts reported in Italy see Cerretti and Tschorsnig (2010). As known, Pn larva hatches from an egg placed on the integument of the victim and, once actively penetrated, consumes its internal organs and kills it (Bagnoli and Lucchi 2006). The existence of the puparium inside the host cocoon tight to the skin of the larva is a distinctive character for the species (Fig. 2F). Though Pn plays an important role in the natural control of *Lobesia botrana*, especially reducing the summer population (Bagnoli and Lucchi 2006, Thiery et al. 2006), it was not considered suitable for the control of *Paralobesia viteana* in the US, because of its relatively low host specificity, the low rate of parasitism reported in nature, and, referring in general to Tachinidae, due to previous experiences of unsuccessful releases (Martinez et al. 2006).

####### Ecological role.

Its importance as parasitoid depends on the host generation; indeed, various authors found that the parasitism rates are more generally related to the EGVM antophagous generation on grapevine: in this case they can overcome 25% of parasitism rate, both on grapevine in Apulia (Laccone 1978) and on *Daphne gnidium* in Sardinia (Luciano et al. 1988) (see table 2). In Tuscany, *Phytomyptera nigrina* (Pn) was mostly found in the vineyards of the medium and lower Arno valley, especially on larvae of the anthophagous generation (Bagnoli and Lucchi 2006). In the natural reserve of San Rossore (Pisa), during several years of investigation carried out on *Daphne gnidium*, a single specimen of Pn was obtained from EGVM larvae of the second generation, collected in late July 2014 (Scaramozzino et al. in press), in contrast to observations carried out on the same host plant by other authors (see Table 3), whereas *Actia pilipennis* was more frequent in our case.

In Piedmont, Pn reached on the first generation of EGVM and EGBM, in two successive years, significant parasitization rates (17.3 and 6.5%), but it was virtually absent (only two individuals obtained) in the second overwintering generation (Colombera et al. 2001).

Silvestri (1912) collected Pn from June to mid-October, Nuzzaci and Triggiani (1982) cited it as the more frequent parasitoid on *Daphne gnidium* in summer, with parasitism rates close to 30%. Laccone (1978) obtained Pn also in the second generation, with significant parasitization rates (from 11.4 to 14.7%). In Veneto, parasitization levels detected for this species were very low in the first generation (0.36 and 0.64%; Marchesini and Dalla Montà 1994), slightly higher, but with a significant 14.6%, in the second generation (Marchesini et al. 2006).

In France, Thiery et al. (2006) found Pn on the first generation of EGVM; they reported parasitization rates ranging from 5.2 to 41.2%. Pn has not been detected for the moment on EGVM overwintering generation, apart from what has been reported in the work of Colombera et al. (2001).

##### Subfamily: Exoristinae

###### Eurysthaea scutellaris

Taxon classificationAnimaliaDipteraTachinidae

(Robineau-Desvoidy, 1848)

Discochaeta hyponomeutae Rond.: Forti 1991 (in Coscollá 1997: 218), Coscollá 1997.

####### Notes.

Coscollà (1997) refers that this tachinid fly is the only parasitoid obtained by Forti (1991) from larvae of EGVM second generation, with a rate of parasitism of 3.7%, though later on Roat and Forti did not mention this species in their list published in 1994. Cerreti and Tschorsnig (2010) reported eight species of Lepidopteran host for *Eurysthaea scutellaris* (mostly Yponomeutidae, but also Tortricidae and Geometridae) for Italy, but he did not mention *Lobesia botrana* among them.

According to Cerreti (2010), Cerreti and Tschornig (2010) and Fauna Europaea, in Italy there are other four species of Tachinidae that could parasitize EGVM, though they have not been found on this host in our country yet (Martinez et al. 2006). These are *Elodia morio* (Fallen, 1820), *Nemorilla floralis* (Fallen, 1810), *Nemorilla maculosa* (Meigen, 1824) and *Pseudoperichaeta nigrolineata* (Walker, 1853) (Martinez et al., 2006).

### Order: HYMENOPTERA

#### Superfamily: ICHNEUMONOIDEA

##### Family: BRACONIDAE

###### Subfamily: Agathidinae

####### Agathis sp.

Taxon classificationAnimaliaHymenopteraBraconidae

Agathis sp. Delrio et al. 1987, Coscollá 1997

######## Italian distribution of reared parasitoids.

Sardinia: Delrio et al. 1987

######## Host range.

The cosmopolitan genus *Agathis* Latreille, 1804, according to Yu (1997–2012) includes 162 species, 35 of which recorded in Europe (Fauna Europaea). They live on larvae of various microlepidoptera, especially Gelechioidea, Pyraloidea and Tortricoidea, as solitary koinobiont endoparasitoid. Detailed information on Agathidinae behavior can be found in Shaw and Huddleston (1991).

######## Ecological role.

In Sardinia Delrio et al. (1987) obtained, by *Lobesia botrana* feeding on grape, an unidentified species of *Agathis* which, in association with other species (*Elachertus affinis* Masi, *Chelonus* sp. and *Habrobracon* sp.), parasitized 10-12% of the first generation larvae and 5% of the second and third generation larvae.

####### Agathis malvacearum

Taxon classificationAnimaliaHymenopteraBraconidae

Latreille, 1805

Agathis malvacearum Moleas 1979, 1995, Coscollá 1997, Hoffman and Michl 2003

######## Italian distribution of reared parasitoids.

Apulia: Moleas 1979, 1995

######## Distribution.

Spread in Central and Southern Europe, UK, Finland, Russia, Caucasus, Turkey, Iran, Central Asia, Canada (Quebec), USA (some States bordering Canada) (Yu et al. 2012).

######## Host range.

*Agathis malvacearum* lives on 7 species of moths: 2 Coleophoridae, 3 Gelechiidae, 1 Pterophoridae and 1 Tortricidae (Yu et al. 2012).

######## Ecological role.

This species was obtained in low numbers during a three-year investigation in vineyards of table grapes in five locations of Apulia and has been associated to EGVM only by Moleas (1979).

####### Bassus linguarius

Taxon classificationAnimaliaHymenopteraBraconidae

(Nees, 1812)

Bassus linguarius Nuzzaci and Triggiani 1982

######## Italian distribution of reared parasitoids.

Apulia: Nuzzaci andTriggiani 1982

######## Distribution.

It occurs in Central and Southern Europe, Great Britain, Finland, Turkey, Iran, Armenia, Kazakhstan and Mongolia (Yu et al. 2012)

######## Host range.

Yu (1997-2012) and Yu et al. (2012) mentions *Coleophora* sp. (Lepidoptera Coleophoridae) as the only known host of this braconid.

######## Ecological role.

In Apulia this species reached 9% of parasitization rate on EGVM larvae developing on *Daphne gnidium* in September.

####### Therophilus tumidulus

Taxon classificationAnimaliaHymenopteraBraconidae

(Nees, 1812)

Microdus tumidulus Nees: Luciano et al. 1988

######## Italian distribution of reared parasitoids.

Sardinia: Luciano et al. 1988

######## Distribution.

*Therophilus tumidulus* is widespread in the Palearctic area: throughout Europe, Morocco, Russia, Caucasus, Turkey, Iran, Central Asia as far as Japan and China (Yu 1997-2012; Yu et al. 2012).

######## Host range.

The species is known as larval parasitoid of Lepidoptera Momphidae, Gelechiidae, Depressariidae and especially Tortricidae, including the vine tortrix moth *Sparganothis pilleriana* (Voukassovitch 1924, Villemant et al. 2012).

######## Ecological role.

*Therophilus tumidulus* was the second most frequent larval parasitoid of EGVM on *Daphne gnidium* in Sardinia, after *Phytomyptera nigrina*, with parasitism rates ranging from 12.5 to 24.1% in the first generation and 8.6% in the third generation. Telenga (1934) mentioned this species as one of the main parasitoids of EGVM in Crimea vineyards.

###### Subfamily: Braconinae

####### Bracon mellitor

Taxon classificationAnimaliaHymenopteraBraconidae

Say, 1836

Bracon mellitor Goidanich 1931
Bracon vernoniae Ashm.: Leonardi 1925

######## Distribution.

This species, distributed in North America from Canada to Mexico, is also present in Cuba, Brazil, Hawaii, while it is not present in Europe. In 1935 it was introduced from Hawaii into Egypt to control the Pink Bollworm, *Pectinophora gossypiella* (Saunders, 1844) (Lepidoptera Gelechiidae), but it seems not established (Bey 1951, Yu et al. 2012).

######## Host range.

*Bracon mellitor* lives on many hosts, mainly belonging to the Coleoptera Curculionidae and several families of Lepidoptera, especially Tortricidae, Pyralidae, Gelechiidae and Noctuidae (Yu 1997-2012; Yu et al. 2012). Among these species are included the already mentioned *Paralobesia viteana* and *Lobesia botrana*. The former was initially confused with *Lobesia botrana* (see e.g. Johnson and Hammar 1912), and it is likely that the record of *Bracon vernoniae* on *Lobesia botrana* reported by Leonardi (1925) originates from this mistake, since Marsh (1979) and Tillman and Cate (1989) did not include this moth among the hosts of this *Bracon*.

####### Bracon (Glabrobracon) admotus

Taxon classificationAnimaliaHymenopteraBraconidae

Papp, 2000

Bracon (Glabrobracon) admotus Loni et al. 2016
Bracon sl.: Scaramozzino et al. (in press)

######## Italian distribution of reared parasitoids.

Tuscany: Loni et al. 2016, Scaramozzino et al. (in press)

######## Distribution.

This species was originally described by Papp (2000) on specimens from Bulgaria and Hungary. Beyarslan and Erdoğan (2012) recorded this species from Turkey, and Loni et al. (2016) from Italy.

######## Host range.

The species was raised from larvae of *Byctiscus betulae* (Linnaeus, 1758) (Coleoptera: Attelabidae) in the leaves of *Populus tremula* L. rolled up like a cigar (Papp 2000)

Loni et al. (2016) obtained three males (two in October 2014 and one in October 2015) by EGVM larvae feeding on *Daphne gnidium* in the Nature Reserve of San Rossore (Pisa).

####### Habrobracon sp.

Taxon classificationAnimaliaHymenopteraBraconidae

Habrobracon sp. Delrio et al. 1987, Moleas 1995, Coscollá 1997

######## Italian distribution of reared parasitoids.

Sardinia: Delrio et al. 1987

######## Host range.

Idiobiont larval ectophagous and gregarious parasitoid predominantly of Coleoptera and Lepidoptera.

######## Ecological role.

In Sardinia vineyards Delrio et al. (1987) obtained by *Lobesia botrana* an unidentified species of *Habrobracon* which, along with other species (*Elachertus affinis* Masi, *Agathis* sp. and *Chelonus* sp.), emerged from 10-12% of the first generation larvae and from 5% of second and third generation larvae. The genus *Habrobracon* Ashmead, 1895 is also used in synonymy with *Bracon* Fabricius, 1804, in the Fauna Europaea, where nearly 250 species of this genus in Europe are listed.

####### Habrobracon concolorans

Taxon classificationAnimaliaHymenopteraBraconidae

(Marshall, 1900)

Habrobracon concolorans Loni et al. 2016
Bracon sl.: Scaramozzino et al. (in press)

######## Italian distribution of reared parasitoids.

Tuscany: Loni et al. 2016, Scaramozzino et al. (in press)

######## Distribution.

*Habrobracon concolorans* is a Trans-Eurasian species (Samartsev and Belokobylskij 2013), widely distributed in the Palaearctic region.

######## Host range.

Loni et al. (2016) found this species associated with EGVM for the first time. In the Nature Reserve of San Rossore, on *Daphne gnidium*, *Habrobracon concolorans* feeds on larvae of the three EGVM generations. It develops as ectoparasitoid on mature larvae, killing them before they make the cocoon, and showing both solitary and gregarious habits, with up to four individuals feeding on the same host larva. To date it is only known from 13 host species, mostly Lepidoptera (Gelechiidae, Noctuidae, Nymphalidae, Pyralidae, Tortricidae) and one Coleoptera Anobiidae (Loni et al. 2016). Moreover, *Habrobracon concolorans* is a major parasitoid of the highly invasive South American tomato leafminer, *Tuta absoluta* (Meyrick, 1917) (Lepidoptera, Gelechiidae) (Biondi et al. 2013).

######## Ecological role.

*Habrobracon concolorans* has been found associated to three other species of Braconinae (*Habrobracon hebetor*, *Habrobracon pillerianae* and *Bracon admotus*) that emerged from more than 1,200 EGVM samples collected in 2014 (Loni et al. 2016) with a parasitization rate of 2.4%.

####### Habrobracon hebetor

Taxon classificationAnimaliaHymenopteraBraconidae

(Say, 1836)

Habrobracon hebetor Moleas 1979, Loni et al. 2016
Habrobracon sp.: Silvestri 1912, Boselli 1928, Stellwaag 1928
Habrobracon brevicornis Wesmael: Goidanich 1934
Microbracon brevicornis Wesm.: Thompson 1946
Bracon sl.: Scaramozzino et al. (in press)

######## Italian distribution of reared parasitoids.

Campania: Silvestri 1912

Tuscany: Loni et al. 2016, Scaramozzino et al. (in press)

Sicily: Silvestri 1912 (from larvae of *Ephestia elutella*)

South Italy: Moleas 1979

######## Distribution.

Cosmopolitan.

######## Taxonomic notes.

In the past, the taxonomic position of *Habrobracon hebetor* was not well defined; it has a large number of synonyms because of the wide distribution, the broad host range and the morphological variability, so that it was attributed to the genera *Bracon*, *Habrobracon* and *Microbracon* (Loni et al. 2016). It was considered for long time separated from his junior synonym *Bracon brevicornis* (Wesmael, 1838) (see e.g.: Marsh 1979, Fauna Europaea) on the basis of various morphological characteristics.

######## Host range.

Highly polyphagous, it is known to attack various species of pyralid moths feeding on stored products, as well as other Lepidopterous pests on several cultivated plants (Yu et al. 2012). It is an idiobiont ectophagous and gregarious parasitoid of Lepidopteran larvae. In Loni et al. (2016) a list of records of *Habrobracon hebetor* found on EGVM is provided. Goidanich (1934), reviewing the specimens obtained from larvae of *Lobesia botrana* and *Ephestia elutella* by Silvestri, assigns them to *Habrobracon brevicornis*. In 2014 Loni et al. (2016) obtained two females of this species from a larva of *Lobesia botrana* feeding on *Daphne gnidium*. Under the name *Habrobracon brevicornis* it was known as a major parasitoid of the European Corn Borer *Ostrinia nubilalis* (Hübner, 1796) (Lepidoptera, Pyralidae), and, with the aim of controlling this pest, it was introduced and released in different locations in North America (Goidanich 1931, Marsh 1979, Yu et al. 2012).

####### Habrobracon pillerianae

Taxon classificationAnimaliaHymenopteraBraconidae

Fischer, 1980

Habrobracon pillerianae Loni et al. 2016
Bracon s. l.: Scaramozzino et al. (in press)

######## Italian distribution of reared parasitoids.

Tuscany: Loni et al. 2016, Scaramozzino et al. (in press)

######## Distribution.

Currently this species is only found in Asian Turkey (Fischer 1980) and in Tuscany (Italy) (Loni et al. 2016)

######## Host range and ecological role.

Very little information is available on this species (Fischer 1980). This author described *Habrobracon pillerianae* on the basis of six specimens emerged from *Sparganothis pilleriana* in Central Anatolia (Turkey). We personally obtained this Braconid by EGVM larvae feeding on grapevine in Cerreto Guidi (FI) in June 2005 and August 2008 and on *Daphne gnidium* in San Rossore (Pisa), from late June to early September 2014 (Loni et al. 2016). Although it proved to be the most common species among the Braconinae found in S. Rossore (it accounted for about 6% of all collected parasitoids), the parasitism rate on *Lobesia botrana* larvae was only around 1.3%.

Also in this species the larvae developed both solitary and gregariously, with up to three individuals feeding on the same host (Loni et al. 2016).

###### Subfamily: Cheloninae

####### Ascogaster quadridentata

Taxon classificationAnimaliaHymenopteraBraconidae

Wesmael, 1835

Fig. 3

Ascogaster quadridentata Luciano et al. 1988, Marchesini and Dalla Montà 1992, 1994, 1998, Coscollá 1997, Marchesini et al. 2006, Bagnoli and Lucchi 2006.

######## Italian distribution of reared parasitoids.

Sardinia: Luciano et al. 1988

Tuscany: Bagnoli and Lucchi 2006

Veneto: Marchesini and Dalla Montà 1992, 1994, 1998, Marchesini et al. 2006

######## Distribution.

The species is present in Europe and North Africa; in Asia it is recorded up to Japan (for more details see: Yu 1997-2012 and Cabi 2016a). *Ascogaster quadridentata* was introduced in North America and New Zealand for the biological control of *Cydia pomonella* L. (Lepidoptera, Tortricidae).

######## Host range.

This koinobiont egg-larval endophagous parasitoid feeds on various species of economically important moths, especially belonging to the family Tortricidae. Yu et al. (2012) provide a list of sixty-seven host species. In the vineyards it has been also associated to *Paralobesia viteana* and *Eupoecilia ambiguella*.

######## Ecological role.

As already highlighted by Bagnoli and Lucchi (2006), in Tuscany this parasitoid is usually present at low density in all the three generations of *Lobesia botrana*. In Veneto it has never been obtained by larvae of the first generation, but reached a maximum rate of parasitism of 4.4% in the second generation and 2.7% in the third generation. In Sardinia it was obtained only from first generation larvae of EGVM living on *Daphne gnidium*, with a parasitism rate of 3.7%.

**Figure 3.**
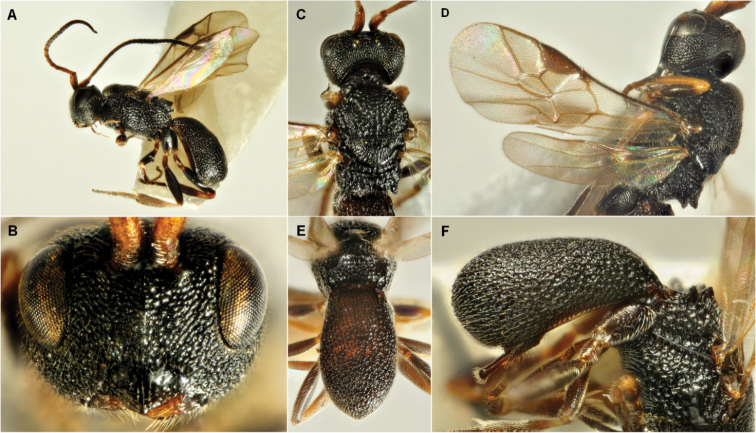
*Ascogaster quadridentata* Wesmael 1835. **A** habitus male, lateral view **B** head male, anterior view **C** head and mesosoma, dorsal view **D** wings, male **E** metasoma male, dorsal view **F** metasoma female lateral view.

####### Ascogaster rufidens

Taxon classificationAnimaliaHymenopteraBraconidae

Wesmael, 1835

Ascogaster rufidens Silvestri 1912, Boselli 1928, Stellwaag 1928

######## Italian distribution of reared parasitoids.

Campania: Silvestri 1912 (Portici)

######## Distribution.

This species shows a Palaearctic distribution, being present in Europe (excluding Iberian Peninsula, ex Yugoslavia and Greece), Russia, Far East Russia, and China.

######## Host range.

Koinobiont endophagous egg-larval parasitoid. The only record is due to Silvestri (1912) that frequently reared it in August from EGVM larvae. Like the previous species, it lives on microlepidoptera, especially Tortricidae.

####### Chelonus sp.

Taxon classificationAnimaliaHymenopteraBraconidae

Chelonus sp. Delrio et al. 1987, Coscollá 1997

######## Italian distribution of reared parasitoids.

Sardinia: Delrio et al. 1987

######## Distribution.

*Chelonus* Panzer, 1806 is a cosmopolitan genus with 190 species in Europe (Fauna Europaea).

######## Host range.

Like the species of the genus *Ascogaster*, the *Chelonus* spp. are koinobiont egg-larval endophagous parasitoids of various groups of microlepidoptera and Noctuidae.

######## Ecological role.

In Sardinia Delrio et al. (1987) obtained an unidentified species of *Chelonus* that, along with other species (*Elachertus affinis* Masi, *Agathis* sp. and *Habrobracon* sp.) parasitized 10-12% of the EGVM larvae of the first generation and 5% of the larvae of the second and third generations.

###### Subfamily: Exothecinae

####### Colastes sp.

Taxon classificationAnimaliaHymenopteraBraconidae

Colastes sp. Colombera at al. 2001

######## Italian distribution of reared parasitoids.

Piedmont: Colombera at al. 2001

######## Distribution and host range.

*Colastes* Haliday, 1833 is a cosmopolitan genus represented in Europe by 15 species, which are, as all the members of the subfamily, idiobiont ectophagous solitary parasitoids on larvae of several leafminers (Shaw and Huddleston 1991). Only one specimen was obtained from the first generation larvae of EGVM in Piedmont.

###### Subfamily: Euphorinae

####### Meteorus sp.

Taxon classificationAnimaliaHymenopteraBraconidae

Meteorus sp. Silvestri 1912, Boselli 1928, Stellwaag 1928

######## Italian distribution of reared parasitoids.

Campania: Silvestri 1912 (Nola, Portici)

Apulia: Silvestri 1912 (S. Vito dei Normanni - Lecce)

######## Distribution.

*Meteorus* Haliday, 1835 is a cosmopolitan genus with a large number of species, Yu (1997-2012) lists 316 species, 46 of which are present in Europe (Fauna Europaea).

######## Host range.

The species of the genus *Meteorus* are koinobiont endophagous larval parasitoids of Coleoptera and Lepidoptera. *Meteorus pendulus* (Müller, 1776) and *Meteorus rubens* (Nees, 1811) have been found on *Eupoecilia ambiguella*, while *Meteorus colon* (Haliday, 1835) was obtained from *Sparganothis pilleriana*. Silvestri (1912) in July and August repeatedly observed some specimens of an unidentified *Meteorus* from larvae of EGVM collected in Campania and Apulia vineyards.

###### Subfamily: Microgastrinae

####### Apanteles sp.

Taxon classificationAnimaliaHymenopteraBraconidae

Apanteles sp. Nuzzaci and Triggiani 1982, Luciano et al. 1988, Moleas 1995, Coscollá 1997

######## Italian distribution of reared parasitoids.

Apulia: Nuzzaci and Triggiani 1982

Sardinia: Luciano et al. 1988

######## Distribution.

*Apanteles* Förster, 1863 is a big cosmopolitan genus which - according to Mason (1981) - would include between 5,000 and 10,000 species. Yu (1997-2012) lists a little less than a thousand species. In Europe are reported 195 species (Fauna Europaea).

######## Taxonomic notes.

*Apanteles* is a polyphyletic complicated group, both for the high number of species and for the evident morphological convergence accompanied by the characters reduction. Mason (1981) divided this group in 26 distinct genera (see Whitfield et al. 2002).

The situation is still controversial and Mason’s opinion is not accepted by all taxonomists of the group (see note 180 in Broad et al. 2012).

######## Host range.

Like all Microgastrinae, *Apanteles* spp. are koinobiont endophagous larval parasitoids of Lepidoptera Ditrysia and are undoubtedly among the most important parasitoids of this order. For more details, see Shaw and Huddleston (1991).

######## Ecological role.

In Apulia, an unidentified species of *Apanteles* was repeatedly found in September-October; this emerged from EGVM larvae living on *Daphne gnidium*, with a parasitization rate of approx. 20% (Nuzzaci and Triggiani 1982). Again, on *Daphne gnidiun* in Sardinia, another unidentified *Apanteles* was obtained both from EGVM larvae of first and third generation, with parasitization rates of 6.2% and 24.1% respectively (Luciano et al. 1988).

####### Apanteles albipennis

Taxon classificationAnimaliaHymenopteraBraconidae

(Nees, 1834)

Apanteles albipennis Laccone 1978

######## Italian distribution of reared parasitoids.

Apulia: Laccone 1978

######## Distribution.

Palaearctic species, widespread in Europe and in the former Soviet Union up to the east coast.

######## Host range.

Yu (1997-2012) provides a list of 33 species of Lepidopteran hosts including Tortricidae, Gelechiidae, Pterophoridae, Coleophoridae, Pyralidae and other families, plus two erroneous records: one species of Buprestidae and one of Curculionidae (Coleoptera). Among the hosts of *Apanteles albipennis* is also recorded *Sparganothis pilleriana* (Ruschka and Fulmek 1915).

######## Ecological role.

Specimens of this species were rarely obtained from EGVM larvae of first and second generation collected on vine in Apulia (Laccone 1978).

####### Microgaster rufipes

Taxon classificationAnimaliaHymenopteraBraconidae

Nees, 1834 [= Microgaster globata auctt., not (Linnaeus, 1758)]

Microgaster globata : Catoni 1914, Stellwaag 1928

######## Italian distribution of reared parasitoids.

Trentino-South Tyrol: Catoni 1914, Stellwaag 1928

######## Distribution.

*Microgaster* is a cosmopolitan genus, fairly rich in species. Yu (1997-2012) reports 178 species, 45 of which in Europe (Fauna Europaea).

######## Taxonomic notes.

In the past, the name “*globata*” was often referred to the European species of *Microgaster* Latreille, 1804, characterized by red hind femora. Nowadays we do not know exactly to what species the old quotes of many authors refer (Nixon 1968). This is probably the case of the records of Catoni (1914) and Schwangart (Stellwaag 1928). Recently Van Achterberg (2014) addressed the issue and eventually renamed *Microgaster globatus* auctt. with the oldest available name *Microgaster rufipes* Nees, 1834.

The species is now reported in the Fauna Europaea as *Microgaster rufipes* Nees, 1834, but is still listed by Broad et al. (2012, 2016) and Yu et al. (2012) under the incorrect name of *Microgaster globata*.

######## Host range.

Yu et al. (2012) list fifty hosts, many of which are tortricids. Those of Catoni (1914) and Schwangart (Stellwaag 1928) are the only references of *Microgaster globata* on *Lobesia botrana*.

####### Microplitis sp.

Taxon classificationAnimaliaHymenopteraBraconidae

Microplitis sp. Marchesini and Dalla Montà 1992, 1994, 1998, Coscollá 1997, Colombera et al. 2001, Marchesini et al. 2006

######## Italian distribution of reared parasitoids.

Piedmont: Colombera et al. 2001

Veneto: Marchesini and Dalla Montà 1992, 1994, 1998, Marchesini et al. 2006

######## Distribution.

*Microplitis* Förster, 1863 is a cosmopolitan genus that counts about 180 species.

######## Host range.

All the species of this genus are solitary or gregarious endoparasitoids of Lepidopteran larvae (especially Noctuidae).

######## Ecological role.

Both Marchesini et al. (1994, 2006) and Colombera et al. (2001) have obtained a few specimens of an unidentified species of *Microplitis* by EGVM larvae of the second generation.

####### Microplitis tuberculifer

Taxon classificationAnimaliaHymenopteraBraconidae

(Wesmael, 1837)

Microplitis tuberculifera : Catoni 1914, Ruschka and Fulmek 1915, Boselli 1928
Microplites tuberculifera (Wesm.) Reinh.: Stellwaag 1928

######## Italian distribution of reared parasitoids.

Trentino-South Tyrol: Catoni 1914, Ruschka and Fulmek 1915

######## Distribution.

*Microplitis tuberculifer* is widespread and common throughout the Palearctic region, with the exception of North Africa.

######## Host range.

It is a solitary koinobiont endoparasitoid of Lepidopteran larvae (Noctuidae and Geometridae), and it is also reported on *Eupoecilia ambiguella* in Austria, together with EGVM (Ruschka and Fulmek 1915), and Bulgaria (Balevski 1989, Yu et al. 2012).

The only Italian records of this species on the two vine moths are due to Catoni (1914) and Ruschka and Fulmek (1915).

###### Subfamily: Rogadinae

####### Aleiodes sp.

Taxon classificationAnimaliaHymenopteraBraconidae

Aleiodes sp. Nuzzaci and Triggiani 1982

######## Italian distribution of reared parasitoids.

Apulia: Nuzzaci and Triggiani 1982

######## Distribution.

*Aleiodes* Wesmael, 1838, is a cosmopolitan genus of 240 species (Yu 1997–2012), with 42 of them listed in Europe (Fauna Europaea).

######## Host range.

In most cases, the species of this genus live on larvae of macrolepidoptera, both diurnal and nocturnal and, to a lesser extent, on larvae of microlepidoptera, including tortricids. They are koinobiont larval endoparasitoids, and lay their eggs in the host young larva and pupate inside the mummified remains of the dead caterpillar. Three species of *Aleiodes* are associated with *Lobesia botrana*. Nuzzaci and Triggiani (1982) obtained a single specimen of an unidentified species of *Aleiodes* by EGVM larvae living on *Daphne gnidium*.

## Supplementary Material

XML Treatment for Actia pilipennis

XML Treatment for Phytomyptera sp.

XML Treatment for Phytomyptera nigrina

XML Treatment for Eurysthaea scutellaris

XML Treatment for Agathis sp.

XML Treatment for Agathis malvacearum

XML Treatment for Bassus linguarius

XML Treatment for Therophilus tumidulus

XML Treatment for Bracon mellitor

XML Treatment for Bracon (Glabrobracon) admotus

XML Treatment for Habrobracon sp.

XML Treatment for Habrobracon concolorans

XML Treatment for Habrobracon hebetor

XML Treatment for Habrobracon pillerianae

XML Treatment for Ascogaster quadridentata

XML Treatment for Ascogaster rufidens

XML Treatment for Chelonus sp.

XML Treatment for Colastes sp.

XML Treatment for Meteorus sp.

XML Treatment for Apanteles sp.

XML Treatment for Apanteles albipennis

XML Treatment for Microgaster rufipes

XML Treatment for Microplitis sp.

XML Treatment for Microplitis tuberculifer

XML Treatment for Aleiodes sp.
